# Angiotensin receptor blockade improves cardiac mitochondrial activity in response to an acute glucose load in obese insulin resistant rats

**DOI:** 10.1016/j.redox.2017.10.005

**Published:** 2017-10-07

**Authors:** Max Thorwald, Ruben Rodriguez, Andrew Lee, Bridget Martinez, Janos Peti-Peterdi, Daisuke Nakano, Akira Nishiyama, Rudy M. Ortiz

**Affiliations:** a School of Natural Sciences, University of California, Merced, USA; b Department of Physiology and Biophysics, Keck School of Medicine, University of Southern California, Los Angeles, CA, USA; c Department of Pharmacology, Kagawa University Medical School, Kagawa, Japan

**Keywords:** Angiotensin II, Mitochondria, Cardiac, Antioxidant enzymes, TCA cycle

## Abstract

Hyperglycemia increases the risk of oxidant overproduction in the heart through activation of a multitude of pathways. Oxidation of mitochondrial enzymes may impair their function resulting in accumulation of intermediates and reverse electron transfer, contributing to mitochondrial dysfunction. Furthermore, the renin-angiotensin system (RAS) becomes inappropriately activated during metabolic syndrome, increasing oxidant production. To combat excess oxidant production, the transcription factor, nuclear factor erythriod-2- related factor 2 (Nrf2), induces expression of many antioxidant genes. We hypothesized that angiotensin II receptor type 1 (AT1) blockade improves mitochondrial function in response to an acute glucose load via upregulation of Nrf2. To address this hypothesis, an oral glucose challenge was performed in three groups prior to dissection (n = 5–8 animals/group/time point) of adult male rats: 1) Long Evans Tokushima Otsuka (LETO; lean strain-control), 2) insulin resistant, obese Otsuka Long Evans Tokushima Fatty (OLETF), and 3) OLETF + angiotensin receptor blocker (ARB; 10 mg olmesartan/kg/d × 6 weeks). Hearts were collected at T0, T60, and T120 minutes post-glucose infusion. ARB increased Nrf2 binding 32% compared to OLETF at T60. Total superoxide dismutase (SOD) and catalase (CAT) activities were increased 45% and 66% respectively in ARB treated animals compared to OLETF. Mitochondrial enzyme activities of aconitase, complex I, and complex II increased by 135%, 33% and 66%, respectively in ARB compared to OLETF. These data demonstrate the protective effects of AT1 blockade on mitochondrial function during the manifestation of insulin resistance suggesting that the inappropriate activation of AT1 during insulin resistance may impair Nrf2 translocation and subsequent antioxidant activities and mitochondrial function.

## Highlights

• ARB increases cardiac mitochondrial enzyme activity in insulin resistant rats.
• Nrf2 binding activity increases when AT1 receptor activation is blocked.
• Glucose suppresses total cardiac GPx and CAT activities during insulin resistance.

## Graphical abstract

**Figure:**
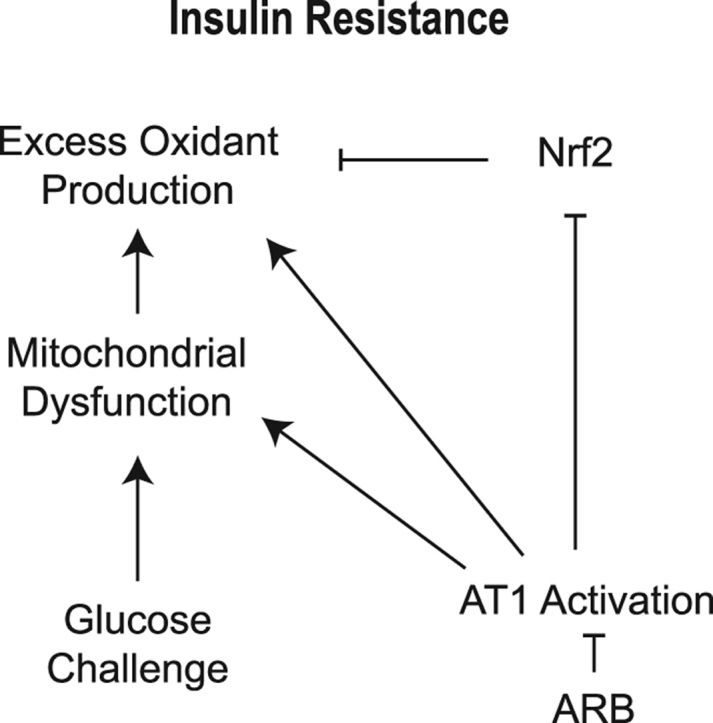

## 1 Introduction

Metabolic syndrome is a rising epidemic in the western world and is characterized by the simultaneous presence of hypertension, dyslipidemia, elevated fasting plasma glucose levels, abdominal obesity, and insulin resistance [11]. Insulin resistance (IR) is a hallmark for the progression of type II diabetes and causes an incomplete uptake of circulating plasma glucose due to impaired insulin secretion and/or receptor signaling [1]. Inappropriate activation of the renin-angiotensin system (RAS) through the angiotensin II type I (AT1) receptor occurs during insulin resistance and has been implicated in contributing to cardiovascular derangements not limited to vasoconstriction, thrombosis, and cardiovascular remodeling [9]. Mitochondrial dysfunction contributes to heart disease, and may contribute disproportionately to the accumulation of oxidative damage during diabetes [2], [19]. Furthermore, cardiomyocytes contain larger amounts of mitochondria compared to other tissues [27], while the heart as a whole contains a lower antioxidant capacity [33] which increases its susceptibility to mitochondria-derived oxidation. In turn, mitochondrial oxidation increases oxidant generation further burdening antioxidant enzymes, which may lessen their ability to correct the oxidant imbalance [14]. Furthermore, mitochondrial and antioxidant dysfunctions may be exacerbated by post-prandial glucose mediated oxidant production in insulin resistant individuals [3], [34]. Among the enzymes that are altered by oxidized conditions are aconitase, and in the citric acid cycle, NADH dehydrogenase (complex I), succinate dehydrogenase (complex II), and cytochrome c reductase (complex III) [4], [22].

Cellular detoxification is an important process that helps remove excess oxidants from the cellular environment through endogenous antioxidants or other molecules capable of reduction. One of the key regulators of antioxidant production is the transcription factor, nuclear factor (erythroid-derived 2)-like 2 (Nrf2), which regulates the production of genes responsible for cellular detoxification among other functions [13]. Nrf2 controls production of several antioxidants such as catalase (CAT), glutathione peroxidase (GPx), and superoxide dismutase (SOD), which together aid in neutralizing excess oxidant production [35]. In order for Nrf2 to translocate to the nucleus, it must dissociate from Kelch-like ECH-associated protein 1 (Keap1) in the cytosol usually by oxidation of Keap1's thiols [7].

The impacts of insulin resistance on the acute changes that glucose imposes on cellular metabolism in the heart are not well described. Evaluation of the heart's mitochondrial response to an acute challenge provides insight on its adaptability and potential to recover from such a constant and frequent insult. This study provides novel information on how cardiac mitochondria respond to a large influx of glucose within two hours post ingestion and the effects of angiotensin receptor blockade (ARB) treatment on the insulin resisitant heart. We hypothesized that AT1 receptor blockade improves cardiac mitochondrial function in response to a glucose load via upregulation of Nrf2.

## 2 Methods

All experimental procedures were reviewed and approved by the institutional animal care and use committees of Kagawa Medical University (Kagawa, Japan), and the University of California, Merced.

### 2.1 Animals

Male, age matched, 10-week-old, lean strain-control Long Evans Tokushima Otsuka (LETO; 279 ± 7 g) and obese Otsuka Long Evans Tokushima Fatty (OLETF; 359 ± 3 g) rats (Japan SLC Inc., Hamamatsu, Japan) were chosen because OLETF rats were previously shown to be insulin resistant at the timeframe chosen for this study [24]. LETO and OLETF rats were assigned to the following groups (n = 5–8 animals/group/time point): 1) untreated LETO, 2) untreated OLETF, and 3) OLETF + angiotensin receptor blocker (ARB; 10 mg olmesartan/kg/d × 6 wk). ARB (Daiichi-Sankyo, Tokyo, Japan) was administered by oral gavage suspended in carboxymethyl cellulose (CMC) to conscious rats. Untreated LETO and OLETF rats were gavaged with CMC only. All animals were maintained in groups of three or four animals per cage in a specific pathogen-free facility under controlled temperature (23 C) and humidity (55%) with a 12-h light, 12-h dark cycle. All animals were given free access to water and standard laboratory rat chow (MF; Oriental Yeast Corp., Tokyo, Japan).

### 2.2 Body mass (BM)

BM was measured on a daily basis to calculate the appropriate amount of ARB to gavage.

### 2.3 Blood pressure

Systolic blood pressure (SBP) was measured at 16 weeks of age in conscious rats by tail-cuff plethysmography (BP-98A; Softron Co., Tokyo, Japan).

### 2.4 Dissections

After 6 weeks of ARB treatment, animals were randomly assigned to 3 different subgroups within each group. Following the subgroup assignment animals were fasted overnight (12 h). The first subgroup of animals was taken following the overnight fast (T0), the second and third subgroups of animals were taken 1 h (T60) and 2 h (T120), following a glucose load (2 g/kg). This protocol allowed us to ascertain the cellular events that transpired in the heart for 2 h post-glucose load. At dissection, animals were decapitated, and trunk blood was collected into chilled vials containing 50 mM EDTA and protease inhibitor cocktail (sigma), and kept on ice until they could be centrifuged. Thereafter the hearts were rapidly removed, weighed, and snap frozen in liquid nitrogen. Frozen samples were kept at −80 C until analyzed.

### 2.5 Western blot analyses

A 35 mg piece of frozen heart was homogenized in 250 μl of Tris-buffered saline containing Triton X-100, SDS, and protease and phosphatase inhibitor cocktail (Sigma). Tissue homogenate was centrifuged (13,200 × *g*, 10 min), and the aqueous layer was transferred to a separate tube and stored at −80 C for later analyses. Membrane fractions were obtained for p47phox translocation using a Minute Plasma Membrane Protein Isolation Kit (Invent Biotechnologies, Plymouth, MN). Membrane fractions were cross-probed with Na^+^/K^+^ ATPase and GAPDH (Santa Cruz Biotechnology, Santa Cruz, CA) antibodies to ensure fraction purity. Total protein content was measured by the Bradford assay (Bio-Rad Laboratories, Hercules, CA). Equal loading of five to forty micrograms of total protein were resolved in 4–15% Tris-HCl SDS gradient gels. Proteins were electroblotted by 2 h wet transfer onto 0.45-μm polyvinyl difluoride membranes. Membranes were blocked with LI-COR Odyssey blocking buffer and incubated for 16 h with primary antibodies (diluted 1:200 to 1:2000) against Keap1 (Santa Cruz Biotechnology, Santa Cruz, CA), AMPK, phosphorylated (p) AMPK at Thr172, Nitrotyrosine (Cell Signaling, Danvers, MA), MnSOD, Cu/ZnSOD (Stressgen, Farmingdale, NY), P47phox and 4HNE (Milipore, Bedford, MA). Membranes were washed, incubated with IRDye 800CW and/or 700CW donkey anti-goat, donkey anti-mouse, or donkey anti-rabbit (LI-COR Biosciences, Lincoln, NE), and rewashed. Blots were visualized using an Odyssey system (LI-COR Biosciences) and quantified using ImageJ. In addition to consistently loading the same amount of total protein per well, the densitometry values were further normalized by correcting with the densitometry values of Ponceau S staining [28].

### 2.6 Biochemical analyses

Nrf2 (Active Motif, Carlsbad, CA), antioxidant enzymes and (CAT, GPx, and SOD) and aconitase (Cayman Chemical, Ann Arbor, MI) activities, and other mitochondrial activities (Succinate Dehydrogenase, and NADH Dehydrogenase) (Abcam, Cambridge, MA) were measured using commercially available kits as previously described [32]. Nrf2 binding was measured using this kit to best assess its binding to the electrophile response element (EpRE). Nuclear fractions were prepared for Nrf2 activity using a NE-PER Nuclear cytosolic extraction kit (Thermo Fisher Scientific, Waltham, MA). Nuclear purity was cross probed with GAPDH and H3 (Cell Signaling, Danvers, MA). Mitochondrial fractions were obtained according to the Aconitase assay instructions. Mitochondrial purity was cross probed using GAPDH and VDAC1 (Abcam, Cambridge, MA). All samples were analyzed in duplicate and run in a single assay with intra-assay and percent coefficients of variability of less than 10% for all assays.

### 2.7 Statistics

Means (± SEM) were compared by two-way ANOVA adjusted for repeated measures to group and time interactions. Pairwise comparisons were made for individual time points. Means were considered significantly different at *P* < 0.05 using Fisher's PLSD. Statistical analyses were performed with the SPSS version 24 software (IBM, Armonk, NY).

## 3 Results

### 3.1 SBP, BM, heart mass, relative heart mass, and glucose tolerance tests

Systolic blood pressure and body mass measurements were taken at the end of the study to observe the status of metabolic syndrome in OLETF rats and to confirm the effectiveness of the ARB. At the end of the study SBP was greater in OLETF compared to LETO by 25% and ARB treatment normalized systolic blood pressure (Table 1). Body mass was greater in OLETF compared to LETO by 37%. ARB treatment had no detectable effect on body mass (Table 1). OLETF heart mass was greater than in LETO by 27%. ARB treatment lowered heart mass in the OLETF rats 13% (Table 1). Relative heart mass was 14% lower in OLETF rats compared to LETO. No detectable differences in relative heart mass was seen with ARB treatment (Table 1). Plasma glucose measurements were taken at dissection to ascertain the degree of insulin intolerance. Fasting plasma glucose was greater in OLETF compared to LETO (106 ± 3 vs. 139 ± 5 mg/dL; p < 0.001) and ARB treatment decreased it compared to OLETF (139 ± 5 vs. 120 ± 2 mg/dL; p < 0.002). At T60 plasma glucose was two-fold in OLETF compared to LETO (154 ± 3 vs. 321 ± 14 mg/dL; p < 0.001) and ARB treatment decreased it compared to OLETF (139 ± 5 vs. 120 ± 2 mg/dL; p < 0.024). At T120 plasma glucose was greater in OLETF compared to LETO (116 ± 1 vs. 168 ± 7 mg/dL; p < 0.001) and ARB treatment decreased it compared to OLETF (168 ± 7 vs. 141 ± 7 mg/dL; p < 0.006).

**Table 1:** Means ± SE SBP, BM, heart mass and relative heart mass.

	LETO	OLETF	OLETF + ARB
Systolic Blood Pressure (mmHg)	114 ± 3	142 ± 2†	120 ± 2*
Body Mass (g)	366 ± 15	503 ± 9†	481 ± 4
Heart Mass (g)	1.03 ± 0.01	1.31 ± 0.02†	1.14 ± 0.02†^,^*
Relative Heart Mass (g/ 100 g BM)	0.28 ± 0.01	0.24 ± 0.01†	0.23 ± 0.01†^,^*

† Significant difference from LETO (P < 0.05).

* Significant difference from OLETF (P < 0.05).

### 3.2 P47phox translocation

**Fig. 1:**
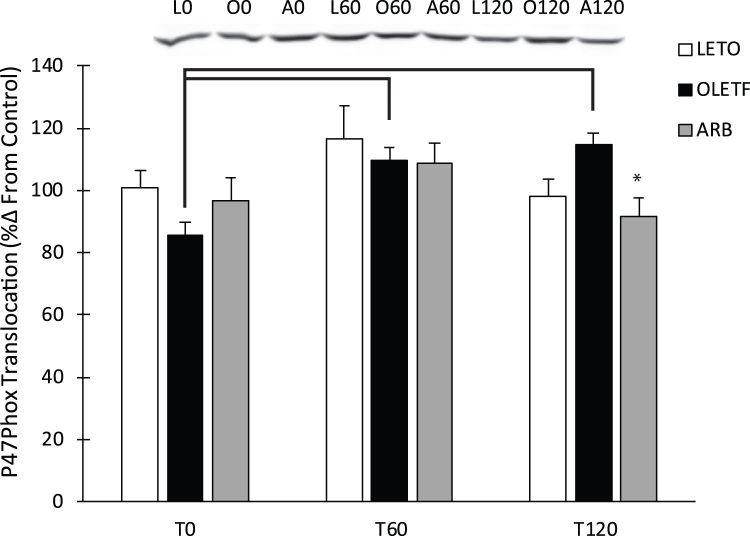
Representative western blot of P47Phox translocation. Means ± SEM values of P47Phox protein expression from Long Evans Tokushima Otsuka (LETO; n = 5), Otsuka Long Evans Tokushima Fatty (OLETF; n = 8), and OLETF + angiotensin receptor type 1 blocker (ARB; n = 8) rats. *Significant difference from OLETF (P < 0.05). Brackets indicate significant differences among respective time points (P < 0.05).

P47phox was measured to assess the contribution of angiotensin II to oxidant production through Nox2 assemblage. Glucose infusion increased the translocation of p47phox 29% over 120 min in OLETF rats. ARB treatment decreased P47phox translocation to the membrane by 22% at 120 min (Fig. 1).

### 3.3 Keap1 & Nrf2

**Fig. 2:**
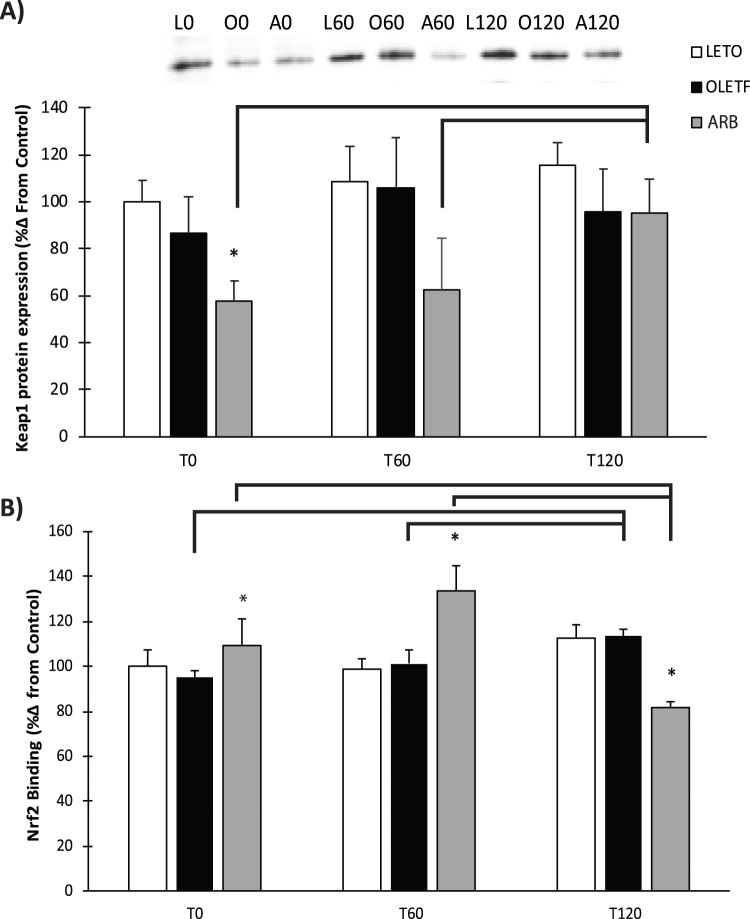
Rssentative western blot of Kelch-like ECH-associated protein 1 and nuclear Nrf2 activity. Means ± SEM values of Keap1 protein expression and nuclear Nrf2 binding from Long Evans Tokushima Otsuka (LETO; n = 5), Otsuka Long Evans Tokushima Fatty (OLETF; n = 8), and OLETF + angiotensin receptor type 1 blocker (ARB; n = 8) rats. *Significant difference from OLETF (P < 0.05). Brackets indicate significant differences among respective time points (P < 0.05). Keap1 had significant independent group and time interactions. Nrf2 has a significant group time interaction.

Keap1 expression levels decreased 54% in ARB compared to OLETF at T0 (Fig. 2**A**). Glucose had no detectable impact on Keap1 expression in LETO or OLETF over the 2 h measurement period; however, Keap1 levels were elevated at T120 by 190% compared to baseline with ARB treatment (Fig. 2**A**). Mean nuclear Nrf2 binding to the EpRE increased 21% at T0 in ARB compared to OLETF, while no change was observed between LETO and OLETF rats (Fig. 2**B**). Glucose infusion increased Nrf2 binding 24% in ARB compared to OLETF at T60 (Fig. 2**B**). Nrf2 levels increased 19% in OLETF rats over the two-hour time frame in response to glucose. Glucose increased Nrf2 binding 22% at T60, while binding decreased 39% at T120 in ARB (Fig. 2**B**).

### 3.4 Mitochondrial protein damage

**Fig. 3:**
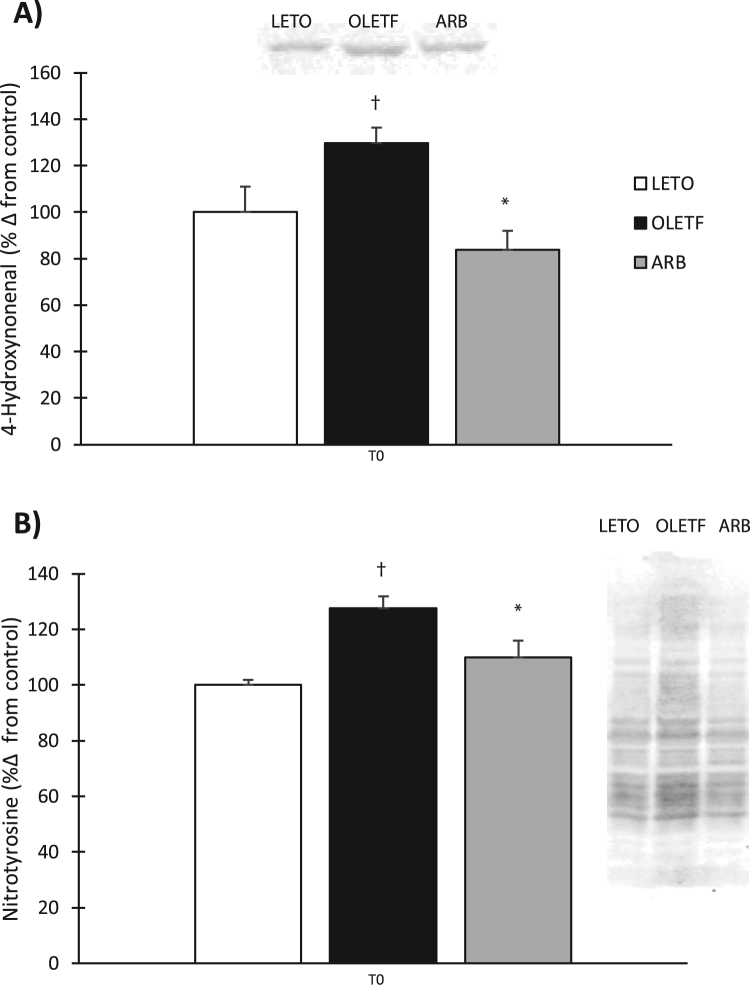
Mitochondrial 4-Hydroxynonenal and Nitrotyrosine measured by western blot. Means ± SEM values of 4HNE protein adducts and nitrated protein from Long Evans Tokushima Otsuka (LETO; n = 5), Otsuka Long Evans Tokushima Fatty (OLETF; n = 8), and OLETF + angiotensin receptor type 1 blocker (ARB; n = 8) rats. †Significant difference from LETO (P < 0.05). *Significant difference from OLETF (P < 0.05).

Indicators of mitochondrial cellular damage were measured to ascertain the amount of damage accumulated in the mitochondria during the progression of insulin resistance, therefore, we measured damage in T0 hearts only. The accumulation of 4-hydroxynonenal (4HNE) adducts increased 30% in OLETF compared to LETO rats (Fig. 3**A**). ARB treated rats had 35% less 4HNE adduct formation compared to OLETF (Fig. 3**A**). The amount of protein nitration increased 28% in OLETF compared to LETO (Fig. 3**B**). ARB treated rats had 14% lower levels of nitrated protein compared to OLETF (Fig. 3**B**).

### 3.5 Antioxidant protein expression and activities

**Fig. 4:**
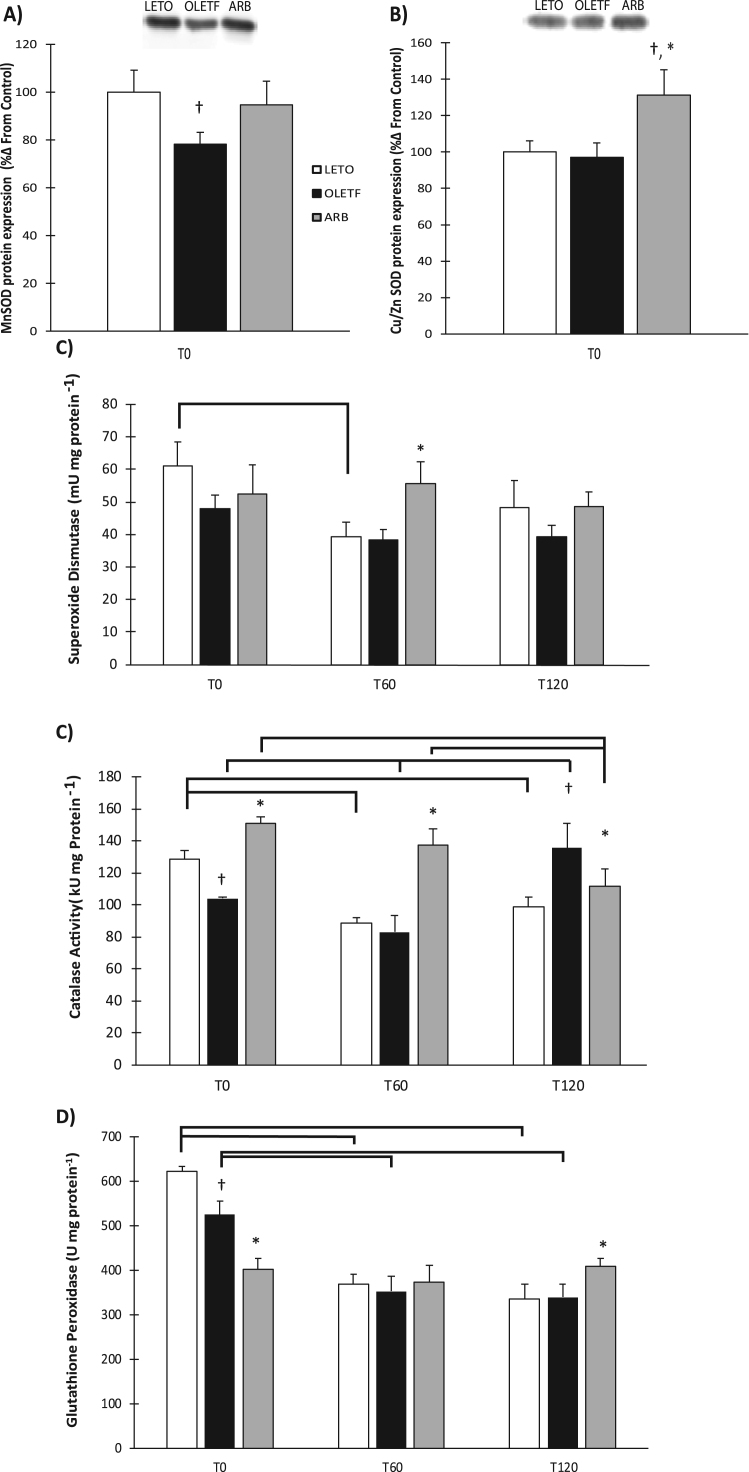
Total cardiac antioxidant protein expression for A) MnSOD B) Cu/ZnSOD and total antioxidant activities for C) superoxide dismutase, D) catalase, and E) glutathione peroxidase. Means ± SEM values of antioxidant activities from Long Evans Tokushima Otsuka (LETO; n = 5), Otsuka Long Evans Tokushima Fatty (OLETF; n = 8), and OLETF + angiotensin receptor type 1 blocker (ARB; n = 8) rats. †Significant difference from LETO (P < 0.05). *Significant difference from OLETF (P < 0.05). Brackets indicate significant differences among respective time points (P < 0.05). Catalase and glutathione peroxidase had significant group time interactions. A significant time interaction as seen with superoxide dismutase.

Protein expression of MnSOD and Cu/ZnSOD were measured at T0 to differentiate which isoform changed in response to ARB treatment. OLETF MnSOD expression decreased 22% compared to LETO (Fig. 4**A**). ARB treated animals increased Cu/ZnSOD expression 31% and 35% compared to LETO and OLETF rats, respectively (Fig. 4**B**). Enzymatic activity of total SOD, Cat, and GPx were assayed to determine the status of various antioxidant enzymes under Nrf2 control during the early stages of insulin resistance and the impact that AT1 receptor blockade has on activity. Glucose suppressed total SOD activity by 36% one hour after administration in LETO rats (Fig. 4**C**). Glucose infusion increased total SOD 31% at T60, in ARB compared to OLETF (Fig. 4**C**). Total CAT activity decreased 19% in OLETF compared to LETO at T0, and levels increased by 44% in ARB compared to OLETF (Fig. 4**D**). No difference occurred at T60 between LETO or OLETF rats, but ARB treatment caused a 40% increase in total CAT activity. Total CAT activity increased 37% in OLETF compared to LETO at T120 and was lowered 17% from OLETF in the ARB treatment group (Fig. 4**D**). Glucose infusion suppressed total CAT activity in LETO by 31% at T60 and 23% at T120 (Fig. 4**D**). Glucose also suppressed total CAT activity in OLETF by 20% at T60 but increased activity by 63% at T120 from T60. Glucose suppressed CAT activity in ARB, but to a much lesser extent (25% by T120 from T0) (Fig. 4**D**). Total GPx activity was 15% lower in OLETF rats compared to LETO at T0. Total GPx activity increased 17% at T120 in ARB compared to OLETF (Fig. 4**E**). Total GPx activity decreased 35% in ARB from LETO at T0 (Fig. 4**E**). Glucose infusion suppressed total GPx activity in LETO and OLETF by 41% and 33%, respectively, at T60, with levels remaining similarly suppressed at T120 (Fig. 4**E**).

### 3.6 Mitochondrial enzyme activities and tissue succinate levels

**Fig. 5:**
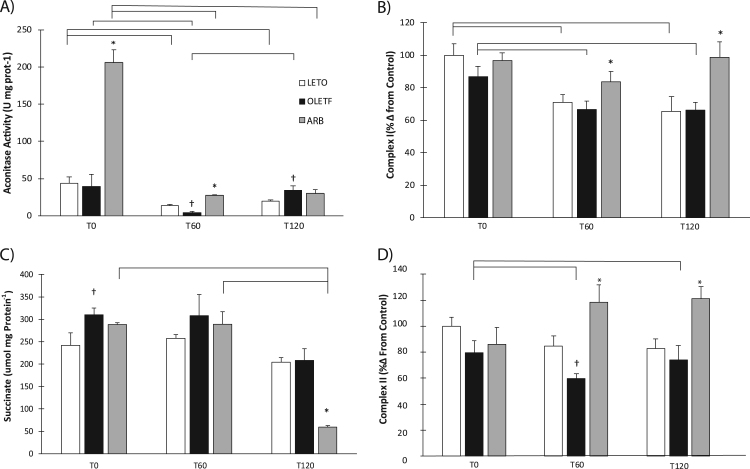
Cardiac mitochondrial activities and succinate tissue content. A) aconitase activity, B) complex I activity, C) tissue succinate content and D) complex II activity. Means ± SEM values of antioxidant activities from Long Evans Tokushima Otsuka (LETO; n = 5), Otsuka Long Evans Tokushima Fatty (OLETF; n = 8), and OLETF + angiotensin receptor type 1 blocker (ARB; n = 8) rats. †Significant difference from LETO (P < 0.05). *Significant difference from OLETF (P < 0.05). Brackets indicate significant differences among respective time points (P < 0.05). Aconitase and succinate had a significant group time interaction. Complex I had significant independent group and time interactions. Complex II had a significant group interaction.

Enzymatic activities of aconitase, complex I, and complex II were measured to assess mitochondrial function in the early stages of insulin resistance. Aconitase activity increased 4-fold over LETO and OLETF at T0 (Fig. 5**A**). At T60 aconitase activity decreased 69% in OLETF compared to LETO. ARB treatment increased levels nearly 6-fold compared to OLETF (Fig. 5**A**). Aconitase activity in OLETF increased 42% compared to LETO at T120. At T60, glucose suppressed aconitase levels in all three groups by 70%, 90% and 87%, respectively, compared to baseline (Fig. 5**A**). By T120 glucose still suppressed aconitase levels by 55%, 14%, and 85%, respectively, compared to baseline (Fig. 5**A**). Complex I exhibited significant group and time interactions independently, but not a group X time interaction. There were no significant differences at baseline; however, ARB treatment increased complex I activity 25% at T60 and 49% at T120 compared to OLETF (Fig. 5**B**). Glucose suppressed complex I activity in both LETO and OLETF rats by 29% and 23%, respectively, at T60 with levels remaining similarly suppressed at T120 (Fig. 5**B**). ARB treatment stabilized complex I activity levels and prevented the suppression at both periods (Fig. 5**B**). Succinate content exhibited a significant group X time interaction with levels increased 22% in OLETF compared to LETO at T0, and ARB was not different from either LETO or OLETF (Fig. 5**C**). Glucose had little effect on succinate content over the two hour period in LETO and OLETF, but did reduce levels in ARB rats by 79% at T120 (Fig. 5**C**). Complex II levels did not differ at baseline; however, glucose suppressed levels in OLETF by 33% at T60 (Fig. 5**D**). ARB treatment increased complex II activity by 76% at T60 (Fig. 5**D**). Complex II levels did not differ between LETO and OLETF at T120, but levels remained 68% higher in ARB compared to OLETF (Fig. 5**D**).

### 3.7 AMPK activation

**Fig. 6:**
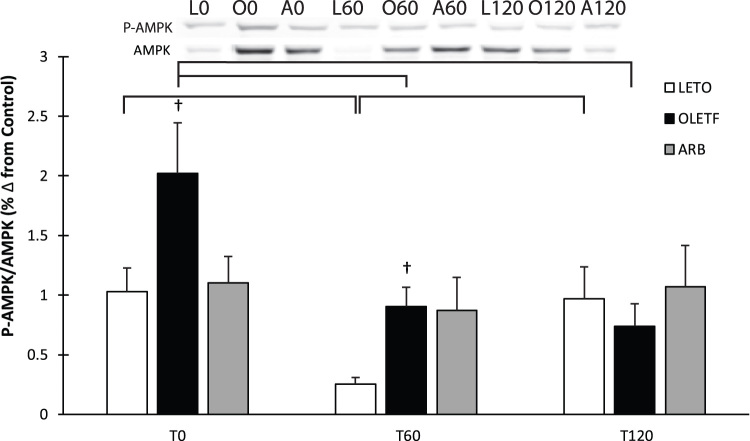
Representative western blot of AMPK and phosphorylated AMPK. Means ± SEM values of P-AMPK/AMPK ratio from Long Evans Tokushima Otsuka (LETO; n = 5), Otsuka Long Evans Tokushima Fatty (OLETF; n = 8), and OLETF + angiotensin receptor type 1 blocker (ARB; n = 8) rats. †Significant difference from LETO (P < 0.05). Brackets indicate significant differences among respective time points (P < 0.05). Significant time interaction.

AMPK expression and phosphorylation (activation) was measured to better assess the impacts of the changes in the mitochondrial enzymes on the energy status of the cell. Activation of AMPK exhibited a significant time interaction with mean AMPK phosphorylation increased two-fold in OLETF at T0 compared to both LETO and ARB (Fig. 6). Glucose suppressed AMPK activation by nearly 80% in LETO and nearly 50% in OLETF at T60, while AMPK phosphorylation remained stable in ARB at both T60 and T120 (Fig. 6). Activation of AMPK returned to baseline levels in LETO at T120, while levels remained the same in OLETF at T120 (Fig. 6).

## 4 Discussion

Some metabolic syndrome features include impaired insulin signaling (insulin resistance), elevated systolic blood pressure, dyslipidemia, and increased adiposity [6], [18]. The factors contributing to the development of insulin resistance are not well known nor is the impact that insulin resistance has on mitochondrial function. However, the inappropriate activation of AT1 has been implicated as a contributor to insulin resistance and mitochondrial dysfunction making it a primary target for study. Furthermore, individuals afflicted with insulin resistance undergo multiple daily bouts of hyperglycemia because of impaired glucose uptake, which may cause cellular damage via oxidant generation from a variety of sources [10]. The aim of this study was to determine the impact of ARB treatment on mitochondrial function and antioxidant activity in the hearts of insulin resistant rats. We found that chronic blockade of AT1 protects and stabilizes mitochondrial enzyme activity in the hearts of insulin resistant rats during an acute glucose challenge.

Inappropriate AT1 activation is a known consequence of metabolic syndrome and increases p47phox translocation, which initiates the assemblage of NADPH oxidase [8], [21]. Exposure to an acute glucose challenge increased p47phox translocation over two hours in OLETF rats, while translocation was stabilized in LETO and ARB with levels decreasing in both groups at the 2-h time point. This indicates that insulin resistance is associated with susceptibility of the heart to abrupt increases in glucose-mediated oxidant production through p47phox translocation which has been implicated in diabetic nephropathy and vascular disease [15], [17]. The ability of chronic AT1 blockade to ameliorate the glucose-induced increase in translocation suggests that this glucose effect is partially AT1 mediated.

Keap1 facilitates degradation of Nrf2 through a Cul3 ring-box ligase [12] and is susceptible to oxidation on its cysteine switches. Oxidation of Keap1 causes liberation of Nrf2 and subsequent translocation in the nucleus. Nrf2, free of Keap1, can regulate phase II gene transcription by binding to the EpRE [26]. Thus, lower Keap1 levels may be indicative of a greater potential of Nrf2 to induced phase II gene transcription, including CAT, GPx, and SOD [29], [35]. The lower Keap1 protein levels in ARB after 6 wks of treatment suggests that the ability of ARB to ameliorate the insulin resistance-associated oxidative damage observed in OLETF rats and other models of diabetes and metabolic syndrome may be achieved by decreasing cytosolic Keap1. Glucose infusion tended to increase Keap1 protein levels in LETO and OLETF over the 2 h; however, levels remained similarly suppressed in ARB at the first measurement hour suggesting that chronic blockade of AT1 protects the heart from any potential glucose-induced increases. Keap1 levels in ARB increased at T120 suggesting that chronic blockade of AT1 desensitizes the heart from abrupt increases in plasma glucose and delays the onset of increasing Keap1. The lack of robust changes in Keap1 protein in LETO and OLETF was associated with similarly unaltered Nrf2 binding to the EpRE. Conversely, the reduced Keap1 levels in ARB were associated with profoundly increased Nrf2 binding, corroborating the impact of AT1 signaling in the Keap1-Nrf2 pathway. Similarly, the increasing trend in Keap1 was associated with decreased Nrf2 binding at T120 in the LETO and OLETF groups. This is important because it demonstrates that Nrf2 activity is not impaired in the early stages of insulin resistance and is not diminished by acutely elevated glucose. This increase in binding is associated with the relative amount of Nrf2 in the nucleus at the respective time points [20].

Aconitase clusters are prone to oxidations and are among the first to undergo a change in transition state during stages of electrophilic stress rendering them inactive [31]. Aconitase activity in LETO and OLETF did not differ at T0 suggesting that the early onset of insulin resistance may not be attributed to robust differences in aconitase activity. However, the decreased activity in OLETF at T60 indicates that the early onset of insulin resistance is associated with impaired glucose handling and likely making the mitochondria susceptible to glucose-induced oxidation and damage. The substantial increase in aconitase activity at T0 in ARB is indicative of the protective effects of chronic AT1 blockade and of a strong association between aconitase activity and AT1 signaling. Despite the dramatic reduction in aconitase activity in ARB following the acute glucose challenge, this level remained substantially greater than those in LETO and OLETF, and was equivalent to their levels at baseline (T0) suggesting that chronic blockade of AT1 helped stabilize the response to a glucose insult.

Enzymes within the electron transport chain such as NADH dehydrogenase (complex I) and succinate dehydrogenase (complex II) are also susceptible to oxidative modification, and it has been proposed that electrons under some circumstances may flow backwards liberating a superoxide radical in the process [22], [23]. Furthermore, succinate levels may increase in metabolically compromised hearts resulting from complex II no longer processing reactions in a forward direction or from oxidation [5]. Furthermore, increased succinate levels in the kidney have been attributed to increasing renin levels, which is the rate limiting enzyme in the formation of Ang II [30]. Chronic AT1 blockade helped stabilize these complex-mediated mitochondrial functions by: 1) ameliorating the susceptibility of complex I activity to an acute glucose insult, and 2) increasing complex II activity. While corresponding changes in heart succinate content and complex II activity levels did not align at the measurement time points, the increased activity in ARB at T60 was followed by substantially reduced succinate content at T120 suggesting that the effects of the changes in enzyme activity on substrate availability are not imparted for at least the following hour in vivo. This suite of mitochondrial measurements demonstrates: 1) the consequences of inappropriate AT1 activation on mitochondrial activity and succinate clearance in the early stages of insulin resistance and metabolic syndrome, and 2) the benefits of chronic AT1 blockade on ameliorating the glucose-induced impairments on mitochondrial function.

SOD, CAT, and GPx are key enzymes responsible for detoxifying oxidants in the cellular environment. Manganese SOD, found in the mitochondria was decreased in OLETF rats while no differences between LETO and OLETF were observed with copper-zinc SOD in the cytosol. However, ARB treatment increased Cu/ZnSOD in the cytosol. In addition to lower expression of MnSOD, increases in mitochondrial 4HNE and NT content was also observed confirming an increase in mitochondrial damage. This suggests that the mitochondria may not be as well equipped to deal with the rapid dismutation of superoxide to hydrogen peroxide during insulin resistance. CAT and GPx are important for removing cellular H_2_O_2_, and thus, detoxifying cells with an oxidizing environment, the decreased activity levels of both in insulin resistant OLETF rats at T0 suggests that the oxidative damage associated with insulin resistance and other metabolic disorders is a consequence of an impaired ability to reduce excess free radical production. Conversely, chronic blockade of AT1 prevented the insulin resistance-associated decrease in CAT activity indicating the impact of activated AT1 signaling on catalase activity in the heart during insulin resistance. Interestingly, the greatest reduction in GPx activity was observed in ARB treated rats; however, we propose that the maintenance of catalase activity may be sufficiently efficient at removing excess H_2_O_2_ to minimize the need for elevated GPx levels. Thus, the increased catalase activity levels may compensate for the reduced GPx levels. Furthermore, glucose either suppressed or tended to suppress antioxidant enzyme activities in LETO and OLETF, but chronic blockade of AT1 consistently prevented this glucose effect highlighting the detriments of inappropriately activated AT1 on redox balance during the manifestation of insulin resistance, and ultimately, metabolic syndrome. Overall, these effects on antioxidant activities imparted by an acute glucose challenge demonstrate how insulin resistance incapacitates the heart from appropriately responding to oxidant generation caused by hyperglycemia, with the consequences being magnified by frequent bouts of exposure to acutely elevated plasma glucose as with a Western diet.

AMPK is a cellular energy sensor that is phosphorylated in response to high intracellular ADP levels to increase GLUT4 translocation into the cell, and subsequently, increase intracellular glucose levels to provide substrate to replenish ATP levels [25]. In metabolic syndrome, sequestration of plasma glucose is impaired through typical insulin signaling, making AMPK-mediated signaling of particular interest as an alternate route for cellular glucose entry [16], [36]. The statically and robustly elevated levels of AMPK phosphorylation (activation) in insulin resistant OLETF rats suggests that increased AMPK phosphorylation may try to compensate for the impaired insulin signaling pathway, and further indication of the metabolic derangement associated with OLETF rats. As expected, the acute glucose challenge suppressed AMPK phosphorylation in LETO and OLETF in the first hour; however, ARB treatment stabilized AMPK activation in response to the glucose, which is a unique observation and demonstrates the importance of stable AT1 signaling in the maintenance of proper glucose regulation.

Hyperglycemia may pose a great deal of stress to the cardiovascular system and these effects may be confounded by preexisting elevations in AT1 activation. In this study, we used an acute glucose challenge in insulin resistant rats with or without ARB treatment to delineate the contributions of AT1 on mitochondrial function in the insulin resistant heart. AT1 activation basally lowered antioxidant activity and increased succinate content. More importantly, AT1 activation when coupled with a surge in glucose levels increased p47phox translocation and decreased mitochondrial enzyme activities including aconitase, complex I, and complex II, which are critical for the proper metabolism of glucose, and when impaired may detrimentally alter substrate metabolism in the insulin resistant heart that may lead to further injury.
