# Supplementary material for: Angiotensin receptor blockade improves cardiac mitochondrial activity in response to an acute glucose load in obese insulin resistant rats
Source: Redox Biol. 2017 Oct 7;14:371–8. doi: 10.1016/j.redox.2017.10.005 (PMC5647524; doi:10.1016/j.redox.2017.10.005)
Supplement: Supplementary file 1 — Supplementary material [file mmc1.docx]

**Table 1: Antibody Details**

| **Antibody Target** | **~ kDa** | **Protein loaded (µg)** | **Primary antibody supplier** | **Host** | **Dilution** | **Time** | **Secondary antibody supplier** | **Host** | **Dilution** | **Time** |
| --- | --- | --- | --- | --- | --- | --- | --- | --- | --- | --- |
| Keap1 | 69kDa | 40ug | Santa Cruz (E-20) Cat# 15246 | Gt | 1 to 200 | O/N | LICOR | DaG | 1 to 25,000 | 1 hr |
| P47Phox | 47kDa | 20ug | Milipore cat# 07-500 | Ms | 1 to 500 | O/N | LICOR | DaM | 1 to 25,000 | 1 hr |
| Ampk | 62kDa | 30ug | Cell Signaling cat#2793s | Rb | 1 to 500 | O/N | LICOR | DaR | 1 to 25,000 | 1 hr |
| Ampk (Phosphorylated) | 62kDa | 30ug | Cell Signaling cat#2531s | Ms | 1 to 2,000 | O/N | LICOR | DaM | 1 to 25,000 | 1 hr |
| MnSOD | 26kDa | 5ug | Stressgen cat#SOD-111 | Rb | 1 to 2,000 | O/N | LICOR | DaR | 1 to 25,000 | 1 hr |
| Cu/ZnSOD | 19kDa | 5ug | Stressgen CAT#SOD-100 | Rb | 1 to 2,000 | O/N | LICOR | DaR | 1 to 25,000 | 1 hr |
| H3 | 17kDa | 5-20ug | Cell Signaling cat#4499s | Rb | 1 to 2,000 | O/N | LICOR | DaR | 1 to 25,000 | 1 hr |
| GAPDH | 37kDa | 2-20ug | Santa Cruz Cat#47724 | Ms | 1 to 500 | O/N | LICOR | DaM | 1 to 25,000 | 1 hr |
| Na+/K+ATPase | 113kDa | 2-6ug | Santa Cruz Cat#48345 | Ms | 1 to 500 | O/N | LICOR | DaM | 1 to 25,000 | 1 hr |
| VDAC1 | 31kDa | 5-15ug | Abcam cat#ab15895 | Rb | 1 to 2,000 | O/N | LICOR | DaR | 1 to 25,000 | 1 hr |
| Nitrotyrosine | Total | 10ug | Cell Signaling cat#9691 | Rb | 1 to 1,000 | O/N | LICOR | DaR | 1 to 25,000 | 1 hr |
| 4HNE | Total | 10ug | Milipore cat#393207 | Rb | 1 to 2,000 | O/N | LICOR | DaR | 1 to 25,000 | 1 hr |

**Validation for Keap1**

**
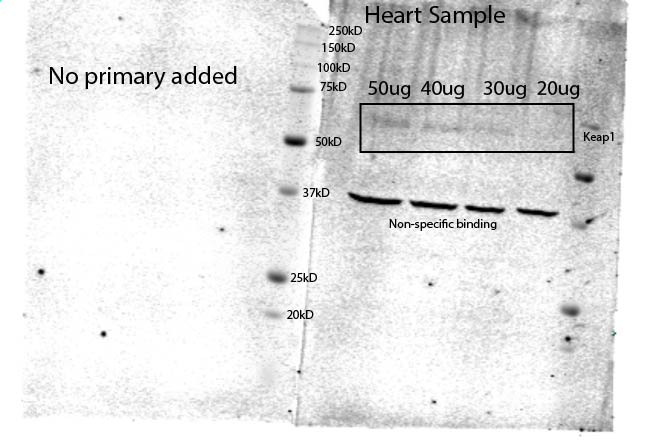
**

**Ponceau S staining for Keap1 (example)**

**
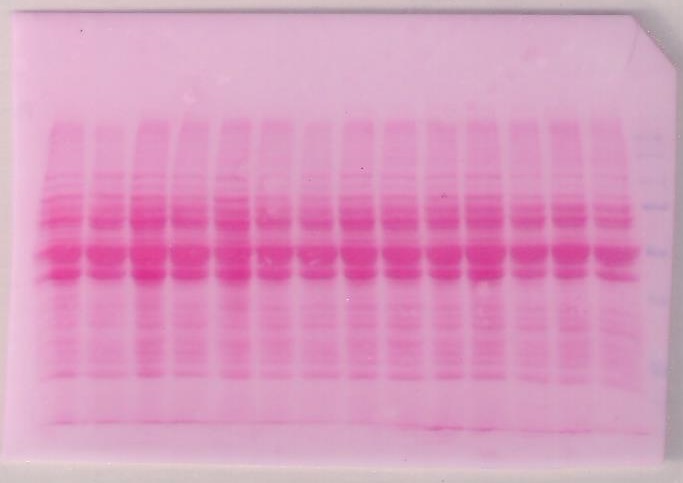
**

**Validation for P47phox (membrane isolation)**

**
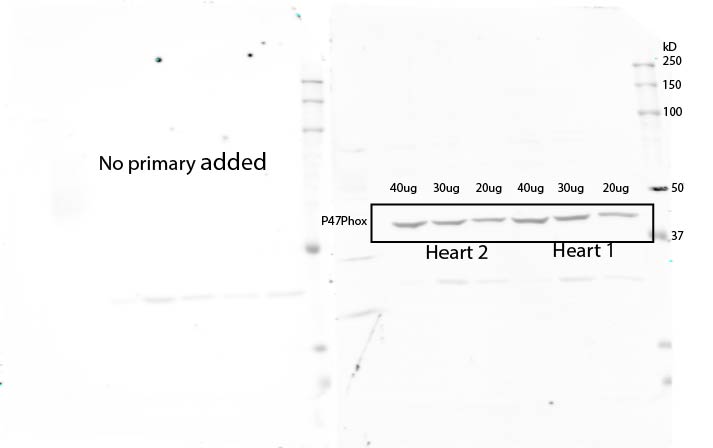
**

**Ponceau S staining for P47phox (example)**

**
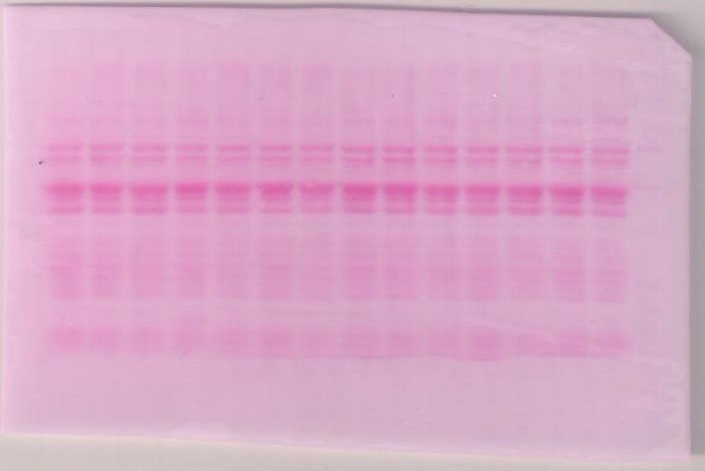
**

**Membrane Purity (Na+/K+ ATPase)**

**
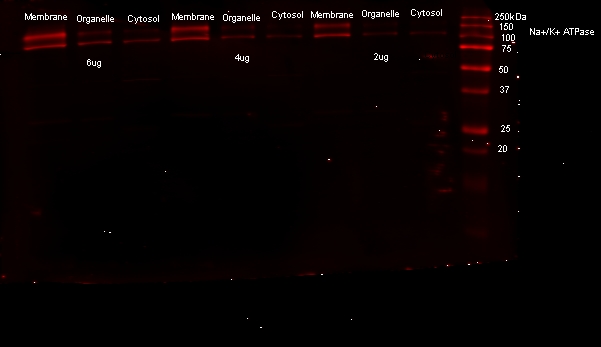
**

**Validation for AMPK (run on same gel as p-AMPK)**

**
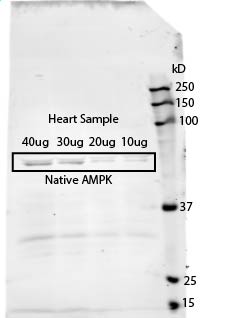
**

**Validation for p-AMPK**

**
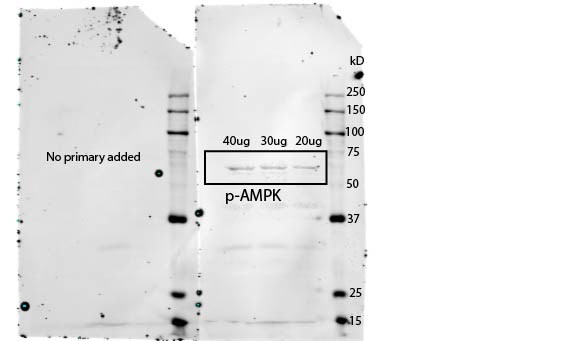
**

**Ponceau staining for p-AMPK/AMPK (example)**

**
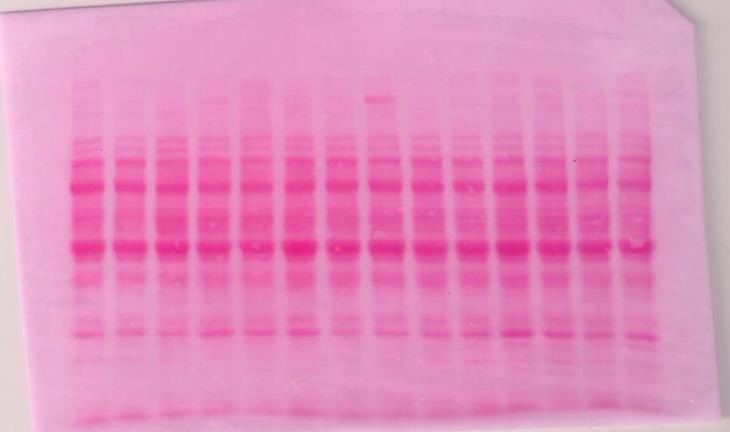
**

Validation for MnSOD

**
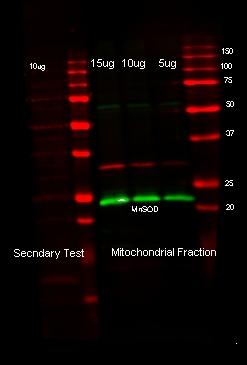
**

**MnSOD Ponceau S stain Loading control**

**
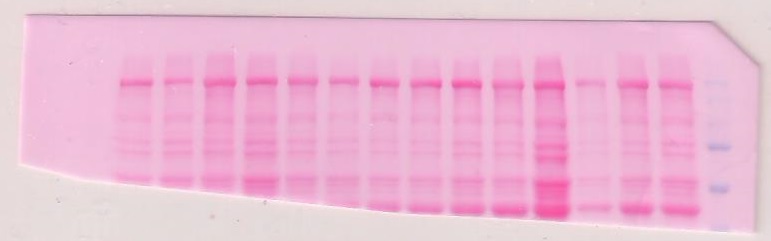
**

**Validation for Cu/ZnSOD**

**
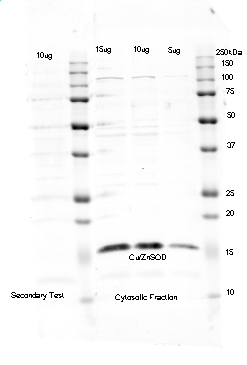
**

**Ponceau S stain for Cu/ZnSOD**

**
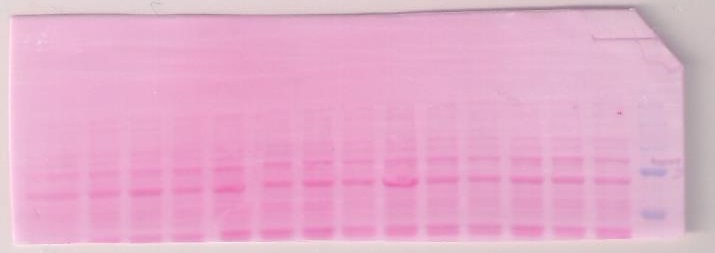
**

**Nuclear/Cytosolic Purity blots (H3-nuclear/GAPDH Cytosolic)**

**
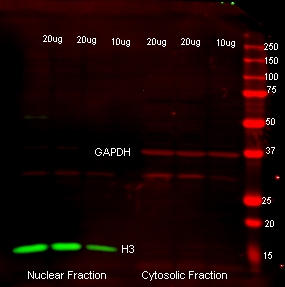
**

**Mitochondrial Purity test (VDAC1 & GAPDH)**

**
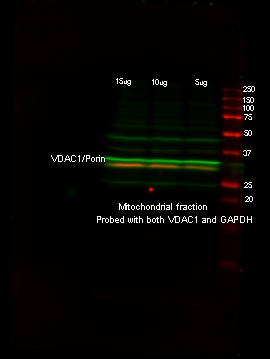
**

**4-hydroxynonenal and Nitrotyrosine Ponceau S stain (Mitochondrial Fraction)**

**
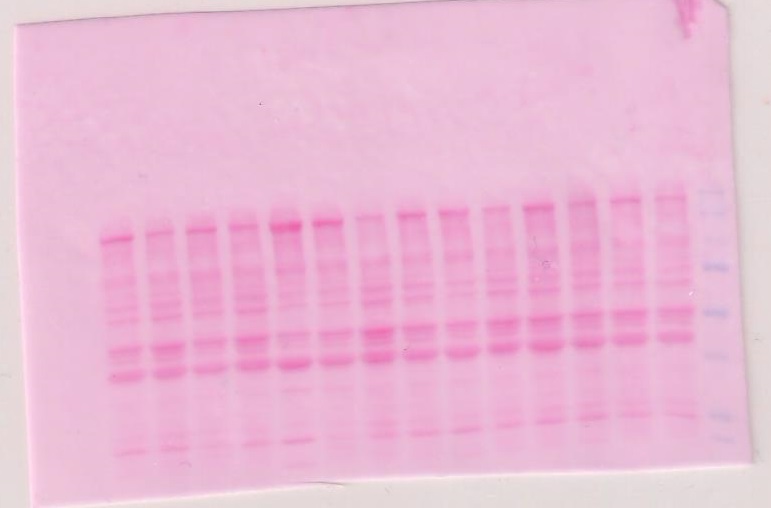
**
